# The experiences of women seeking help for vaginismus and its impact on their sense of self: An integrative review

**DOI:** 10.1177/17455057231199383

**Published:** 2023-09-28

**Authors:** Rashmi Pithavadian, Jane Chalmers, Tinashe Dune

**Affiliations:** 1 School of Health Sciences, Western Sydney University, Penrith, NSW, Australia; 2 Allied Health and Human Performance, University of South Australia, Adelaide, SA, Australia; 3 Translational Health Research Institute, Western Sydney University, Penrith, NSW, Australia; 4 Psychological Science at Australian College of Applied Professions, Sydney, NSW, Australia

**Keywords:** vaginismus, genito-pelvic pain/penetration disorder, female sexual dysfunction, painful sex, help-seeking, treatment-seeking, sense of self

## Abstract

**Background:**

There is low social awareness of vaginismus despite it being a relatively common female sexual dysfunction that makes vaginal penetration painful, difficult, and/or impossible. While existing literature on vaginismus has had a clinical focus on the affected genitalia, there is a lack of research on women’s help-seeking experiences of vaginismus from their perspective.

**Objectives:**

This integrative review’s objective was to explore: women’s help-seeking experiences of vaginismus, and how such help-seeking experiences impact their sense of self.

**Design:**

Cooper’s five-step integrative review approach was followed to develop a research question, a search strategy, selection criteria, and data evaluation, analysis, and presentation.

**Data Sources and Methods:**

A systematic search of the literature was completed in the following seven databases in January 2023: PsycINFO, ProQuest Central, PubMed, Scopus, CINAHL, Cochrane, and Embase. Out of the 373 articles found through database searches and additional citation searching, 22 studies were included in this review for meeting the eligibility criteria of having an empirical design, being written in English, and examining women’s help-seeking experiences for vaginismus and its impact on their sense of self.

**Results:**

Thematic analysis was used to summarize the findings from the included studies which were informed by 1671 participants. *Help-Seeking Process, Medical Management, Help-Seeking and Sense of Self*, and *Holistic Care Recommendations from the Findings* emerged as four major themes with corresponding subthemes.

**Conclusion:**

This review indicates that women continue to face difficulties in seeking and receiving help for vaginismus even through the healthcare system. However, the studies did not explicitly discuss how women’s help-seeking for vaginismus impacted their sense of self. This highlights an epistemological gap on how women’s help-seeking for their vaginismus impacts their sense of self, which can affect their treatment responses. Recommendations are provided for future healthcare and research to improve health outcomes for women with vaginismus.

## Introduction

### Vaginismus

Vaginismus is characterized by the persistent involuntary closure of the vagina upon attempts at penetration of any sort whether by a penis, tampons, fingers, or speculum.^1^ This can make penetration via vaginal entry painful, difficult, and/or impossible depending on the severity of vaginismus.^2^ Vaginismus can be diagnosed as primary (lifelong) or secondary (acquired).^3^ The current fifth edition of the Diagnostic and Statistical Manual controversially merged vaginismus and dyspareunia into genito-pelvic pain/penetration disorder (GPPPD).^4,5^ While some believed that this merging would improve diagnostic accuracy, others continue to argue that the scope of GPPPD is too broad and does not capture the clinical difference between dyspareunia and vaginismus.^5–7^ Dyspareunia is often used as a general term that refers to painful sexual intercourse due to varied causes such as endometriosis, vulvodynia, or vaginal atrophy to name a few.^8^ The term dyspareunia does not capture the specific combination of symptoms of pain, anxiety, and excessive vaginal tightness or closure upon attempts at vaginal penetration that occurs with vaginismus or the treatment for it. Also, the term vaginismus continues to be used in clinical settings to diagnose patients and in recent research.^6,9,10^ The authors recognize the unique experiences of vaginismus and not the dyspareunic conditions it is merged with in GPPPD. Vaginismus will therefore be used to refer to the condition throughout this article.

Due to making sexual intercourse or any type of vaginal sexual penetration difficult or unachievable, vaginismus is regarded as a female sexual dysfunction or female sexual pain disorder. Yet, vaginismus remains a specialist term that never transitioned into common usage in the same way as erectile dysfunction and premature ejaculation.^11^ This contributes to the low social awareness of vaginismus throughout societies around the world. While there has been some increased interest in vaginismus, it is still a specialist term that is not known well enough in the public to be casually mentioned in social circles, movies, or television in the same way as erectile dysfunction or premature ejaculation. Vaginismus continues to be under-researched^6,12^ and not well known among women and even among health professionals.^2,6,12^

### Current research on vaginismus and its prevalence

Vaginismus is reported to be a common female sexual dysfunction.^13–15^ Yet, there is no consensus on the specific prevalence of vaginismus.^6,14,15^ A recent study from Denmark assessed the prevalence of vaginismus as affecting 0.8% of the female population.^16^ However, different studies have estimated vaginismus to affect anywhere between 7% and 68% of the female population with higher reported prevalence in certain countries, such as Iran, Turkey, and Ghana.^6,12,14,17,18^ McEvoy et al.^6^ highlight that information on prevalence of vaginismus is only based on women who attend clinics. However, due to the guilt, shame, and secrecy of vaginismus, many with the condition do not seek help and are therefore not counted.^6^ This makes it difficult to estimate the general prevalence of the condition.^6^ Given that this indicates that many women suffer with vaginismus, it is important to examine women’s experiences of making sense of the condition.

However, the existing literature on vaginismus has had a large clinical focus on the affected genitalia in terms of aetiology,^19,20^ efficacy of treatments,^21–23^ and ongoing debates regarding the classification and diagnostic criteria of vaginismus.^5,24^ Many studies that examine fear, anxiety, or disgust in relation to vaginismus still have a clinical focus on how it affects women’s response to penetration rather than their phenomenological experiences of it from their perspective. Sánchez Bravo et al.’s^25^ Spanish-written work examines how conflict between desire and sexual prohibition causes associations of sex with pain. Koops et al.’s^26^ quantitative study also considers personality functioning in relation to vaginismus symptoms. In a recent systematic review by Mellado et al.^27^ that included 52 studies on female pelvic pain conditions, only two qualitative studies examining the impact of vaginismus and treatment on women’s lives were identified in the literature. This indicates a lack of research on women’s subjective experiences of struggling with vaginismus from their perspective.

### Help-seeking

Women with female sexual dysfunctions face barriers to seek help and access treatment.^28^ In health contexts, help-seeking behaviour can be understood as people’s process, interactions and experiences to search for help to gain diagnosis, information, and treatment or relief for a presenting health issue.^29^ Moreover, the healthcare system has shown to be androcentric and dismissive of women’s sexual problems when they seek help.^30^ This increases the risk of women with vaginismus being misdiagnosed, given inappropriate treatment, or having their experience dismissed altogether. Not receiving help for vaginismus can have severe consequences on women’s relationships, sex life, and emotional wellbeing, which can snowball to cause mental health issues and overall poor quality of life.^31,32^

### Sense of self

Women face challenges to seek help to gain information, diagnosis, and treatment for taboo conditions such as vaginismus,^33^ which can impact their sense of self. The concept of sense of self refers to how one perceives themselves as an individual.^34^ Sense of self can be understood as an umbrella term that can include self-esteem, self-awareness, and self-reflection to name a few. For clarity in expression, ‘sense of self’, ‘construction/s of self’, and ‘self’ will be used interchangeably in this article. The body, and its afflictions such as vaginismus, influence one’s sense of self.^34,35^ Women’s social interactions to seek help through the healthcare system and other avenues can affect their sense of self too.^35^ Therefore, women’s constructions of self are unique to their individual social interactions, which renders sense of self to be a highly subjective phenomenon that is difficult to quantitively measure. Understanding how help-seeking for vaginismus impacts women’s sense of self can reveal valuable insight into women’s processes to make sense of negotiating treatment for the condition.

### Objective

The experiences of women help-seeking for their vaginismus can impact their constructions of self in ways that influence how well they perceive and respond to interventions. An integrative review, which can include diverse methods of research, was therefore employed to examine such broad phenomena of experience.^36^ This mixed-methods integrated review was undertaken as the first study to summarize the existing literature on how women seek help for vaginismus, and its impact on their constructions of sense of self. Using a Population, Phenomena of Interest, and Context (PICo) framework,^37^ an integrative literature review was conducted to answer the following question: What are women’s experiences to seek help for their vaginismus, and how does such help-seeking impact their sense of self?

## Methods

This was an integrative review using Cooper’s^38,39^ five-stage framework. This review followed the five stages of (1) formulating the purpose and research question(s) which has been outlined above, (2) systematic literature search and selection, (3) data evaluation, (4) data analysis, and (5) data presentation.^38,39^ As a mixed-methods review, it is able to integrate quantitative evidence of effectiveness and qualitative data on participants’ experiences.^40^ This can offer a broader and deeper understanding of the complex issue of women’s help-seeking experiences for their vaginismus and its impact on their constructions of self. Estimating the effectiveness of an intervention from included studies is not applicable in integrative reviews which examine broader experiential phenomena.

### Search strategy

Unlike systematic reviews, integrative review guidelines have not been formalized for literature searches.^41^ Therefore, to support stage two of Cooper’s framework, the Preferred Reporting Items for Systematic Reviews and Meta-Analyses (PRISMA) guidelines^42,43^ were adapted to report strategies to search, screen, and select literature as suggested.^41^ The literature search included seven databases: PsycINFO, ProQuest Central, PubMed, Scopus, CINAHL, Cochrane, and Embase. The eligibility criteria presented in Table 1 were devised by all authors to translate the review’s research question into searchable keywords to screen the database results step-by-step by title, abstract, and full-text.^42^ The Medical Subject Heading (MeSH) terms searched on databases are also presented in Table 1. Relevant keywords, Boolean phrases, advanced search techniques of using truncated symbols, MeSH terms, and any applicable subject headings were first searched on all seven databases in English in January 2023 which produced 373 results as detailed in Table 2. There were 87 duplicates that were removed to leave 286 results which were systematically screened by title, abstract, and full-text by the first two authors. Five eligible studies were found in the search.

**Table 1.** Summary of the inclusion/exclusion criteria and keywords.

PICo parameters		Inclusion	Exclusion	Key words/steps	MeSH terms searched on applicable databases
Population (P)		Literature which focuses on women with vaginismus	Literature which does not focus on women with vaginismus. Other dyspareunic conditions which are not vaginismus. Empirical studies on combined female sexual dysfunctions where participants with vaginismus were not separated to extract relevant data.	(Title) (vaginismus OR dyspareunia OR vaginism OR ‘genito-pelvic pain/penetration disorder’ OR GPPPD OR ‘penetration disorder’ OR ‘vaginal pain’)	Vaginismus, Dyspareunia
Phenomena of interest (I)		Literature which mentions people’s help-seeking for their vaginismus and its impact on constructions of sense of self in the abstract	Literature which does not mention people’s help-seeking for their vaginismus and its impact on constructions of self in the abstract	AND (Abstract) (‘help-seeking’ OR help-seeking OR treatment OR ‘treatment-seeking’ OR treatment-seeking OR diagnosis OR information OR relief OR support) AND (Abstract) (experience OR perception OR construct* OR perceiv*) AND (Abstract) (self* OR identity)	Help-seeking behaviour OR Information-seeking behaviour AND Self Concept OR Gender Identity
Context (Co)	Location	International	None	N/A	N/A
	Language	English	Other languages	English	English
	Time	Any	None	N/A	N/A
Study/literature type		Published peer-reviewed primary research including qualitative, quantitative, or mixed-method designs	Published literature which DOES NOT include qualitative, quantitative, or mixed methods of data collection and analysis	N/A	N/A

GPPPD: genito-pelvic pain/penetration disorder.

**Table 2.** Database searches.

PsycINFO (EBSCO)	S1	TI vaginismus OR dyspareunia OR vaginism OR “genito-pelvic pain/penetration disorder” OR GPPPD OR “penetration disorder” OR “vaginal pain”	306
	S2	MM “Vaginismus”	216
	S3	MM “Dyspareunia”	253
	S4	AB “help seeking” OR help-seeking OR treatment OR “treatment seeking” OR treatment-seeking OR diagnosis OR information OR relief OR support	1,647,840
	S5	MM “Help Seeking Behavior” OR MM “Health Care Seeking Behavior”	9519
	S6	MM “Health Care Seeking Behavior” OR MM “Professional Referral” OR MM “Self-Referral”	7147
	S7	MM “Information Seeking” OR MM “Questioning”	6056
	S8	AB experience OR perception OR construct* OR perceiv*	1,197,401
	S9	MM “Self-Perception” OR MM “Belonging” OR MM “Body Image” OR MM “Interoception” OR MM “Perceived Control” OR MM “Self-Acceptance” OR MM “Self-Deception” OR MM “Self-Efficacy” OR MM “Self-Knowledge” OR MM “Self-Reference” OR MM “Self-Reflection”	52,927
	S10	AB self* OR identity	711,408
	S11	MM “Self-Concept” OR MM “Academic Self Concept” OR MM “Athletic Identity” OR MM “Entitlement (Psychological)” OR MM “Impostor Phenomenon” OR MM “Professional Identity” OR MM “Self-Compassion” OR MM “Self-Confidence” OR MM “Self-Congruence” OR MM “Self-Esteem” OR MM “Self-Forgiveness” OR MM “Self-Regard” OR MM “Self-Worth” OR MM “Sense of Coherence”	67,184
	S12	MM “Gender Identity” OR MM “Cisgender” OR MM “Gender Nonbinary” OR MM “Gender Nonconforming” OR MM “LGBTQ” OR MM “Transsexualism”	12,815
	S13	S1 OR S2 OR S3	458
	S14	S4 OR S5 OR S6 OR S7	1,653,656
	S15	S8 OR S9	1,225,192
	S16	S10 OR S11 OR S12	731,000
	S17	S13 AND S14 AND S15 AND S16	25
	S18	S13 AND S14 AND S15 AND S16 Narrow by Language: English	**25**
CINAHL	S1	(MM “Vaginismus”)	72
	S2	(MM “Dyspareunia”)	509
	S3	TI vaginismus OR dyspareunia OR vaginism OR “genito-pelvic pain/penetration disorder” OR GPPPD OR “penetration disorder” OR “vaginal pain”	366
	S4	AB “help seeking” OR help-seeking OR treatment OR “treatment seeking” OR treatment-seeking OR diagnosis OR information OR relief OR support	1,741,128
	S5	(MM “Help Seeking Behaviour”)	4270
	S6	(MM “Information Seeking Behaviour”)	2777
	S7	AB experience OR perception OR construct* OR perceiv*	607,652
	S8	AB self* OR identity	328,534
	S9	(MM “Self Concept +”)	31,731
	S10	(MM “Gender Identity +”)	4806
	S11	S1 OR S2 OR S3	639
	S12	S4 OR S5 OR S6	1,743,692
	S13	S8 OR S9 OR S10	343,943
	S14	S11 AND S12 AND S7 AND S13	**17**
Cochrane	#1	MeSH descriptor: [Vaginismus] explode all trees	13
	#2	MeSH descriptor: [Dyspareunia] explode all trees	233
	#3	(vaginismus OR dyspareunia OR vaginism OR “genito-pelvic pain/penetration disorder” OR “penetration disorder” OR gpppd OR “vaginal pain”): ti (Word variations have been searched)	7667
	#4	MeSH descriptor: [Help-Seeking Behaviour] explode all trees	47
	#5	MeSH descriptor: [Information-Seeking Behaviour] explode all trees	54
	#6	(“Help seeking” OR help-seeking OR treatment OR treatment-seeking OR “treatment seeking” OR diagnosis OR information OR relief OR support): ab (Word variations have been searched)	928,439
	#7	(experience OR perception OR construct* OR perceiv*): ab (Word variations have been searched)	142,266
	#8	(self* OR identity): ab (Word variations have been searched)	134,527
	#9	MeSH descriptor: [Gender Identity] explode all trees	279
	#10	MeSH descriptor: [Self Concept] explode all trees	7704
	#11	#1 OR #2 OR #3	7804
	#12	#4 OR #5 OR #6	928,448
	#13	#8 OR #9 OR #10	136,810
	#14	#11 AND #12 AND #7 AND #13	**42**
Embase	1	vaginismus.mp. or exp *vaginism/	982
	2	Limit 1 to English language	809
	3	dyspareunia.mp. or exp *dyspareunia/	14,203
	4	Limit 3 to English language	13,122
	5	vaginism.mp. or exp *vaginism/	1245
	6	Limit 5 to English language	1064
	7	“genito-pelvic pain/penetration disorder”.mp.	72
	8	Limit 7 to English language	70
	9	GPPPD.mp.	22
	10	Limit 9 to English language	21
	11	“penetration disorder”.mp.	116
	12	Limit 11 to English language	113
	13	“vaginal pain”.mp. or exp *vagina pain/	577
	14	Limit 13 to English language	557
	15	2 or 4 or 6 or 8 or 10 or 12 or 14	14,163
	16	exp *help seeking behaviour/	3840
	17	Limit 16 to English language	3771
	18	information seeking.mp. or exp *information seeking/	6953
	19	Limit 18 to English language	6791
	20	“treatment seeking”.mp.	8036
	21	Limit 20 to English language	7971
	22	treatment-seeking.mp.	8036
	23	Limit 22 to English language	7971
	24	“help seeking”.mp.	20,704
	25	Limit 24 to English language	20,305
	26	“help-seeking”.mp.	20,704
	27	Limit 26 to English language	20,305
	28	treatment.mp.	8,585,885
	29	Limit 28 to English language	7,508,193
	30	diagnosis.mp. or exp *diagnosis/	7,555,104
	31	Limit 30 to English language	6,208,544
	32	exp *information/ or information.mp.	2,985,312
	33	Limit 32 to English language	2,793,728
	34	relief.mp.	157,636
	35	Limit 34 to English language	143,496
	36	support.mp.	1,692,333
	37	Limit 36 to English language	1,624,428
	38	17 or 19 or 21 or 23 or 25 or 27 or 29 or 31 or 33 or 35 or 37	14,829,902
	39	experience.mp. or exp *experience/	1,256,428
	40	Limit 39 to English language	1,121,815
	41	perception.mp. or exp *perception/	513,729
	42	Limit 41 to English language	483,311
	43	perceiv*.mp.	355,489
	44	Limit 43 to English language	345,823
	45	construct*.mp.	803,148
	46	Limit 45 to English language	752,192
	47	40 or 42 or 44 or 46	2,484,754
	48	self.mp. or exp *self concept/	1,325,403
	49	Limit 48 to English language	1,259,422
	50	exp *gender identity/ or exp *identity/ or identity.mp.	208,392
	51	Limit 50 to English language	199,104
	52	49 or 51	1,425,375
	53	15 and 38 and 47 and 52	**230**
ProQuest	S1	(MJMESH.EXACT.EXPLODE(“Vaginismus: C.12.100.250.919”) OR MJMESH.EXACT.EXPLODE(“Vaginismus: C.12.050.351.500.894.870”) OR MJMESH.EXACT.EXPLODE(“Vaginismus: C.12.100.875.871”) OR MJMESH.EXACT.EXPLODE(“Vaginismus: C.12.100.250.894.870”) OR MJMESH.EXACT.EXPLODE(“Vaginismus: F.03.835.900”) OR MJMESH.EXACT.EXPLODE(“Vaginismus: C.12.050.351.500.919”)) OR (MJMESH.EXACT.EXPLODE(“Dyspareunia: F.03.835.199”) OR MJMESH.EXACT.EXPLODE(“Dyspareunia: C.12.100.875.242”) OR MJMESH.EXACT.EXPLODE(“Dyspareunia: C.12.100.250.110”) OR MJMESH.EXACT.EXPLODE(“Dyspareunia: C.12.100.500.100”) OR MJMESH.EXACT.EXPLODE(“Dyspareunia: C.12.200.294.100”) OR MJMESH.EXACT.EXPLODE(“Dyspareunia: C.12.050.351.500.110”)) OR ti(vaginismus OR dyspareunia OR vaginism OR “genito-pelvic pain/penetration disorder” OR GPPPD OR “penetration disorder” OR “vaginal pain”) *(Limit to peer reviewed applied)*	356
	S2	ab(“help seeking” OR help-seeking OR treatment OR “treatment seeking” OR treatment-seeking OR diagnosis OR information OR relief OR support) OR MJMESH.EXACT.EXPLODE(“Help-Seeking Behaviour”) OR (MJMESH.EXACT.EXPLODE(“Information Seeking Behaviour: L.01.143.458”) OR MJMESH.EXACT.EXPLODE(“Information Seeking Behaviour: F.01.145.209.372”) OR MJMESH.EXACT.EXPLODE(“Information Seeking Behaviour: F.01.145.535”)) OR MAINSUBJECT.EXACT(“Health behaviour”) OR MAINSUBJECT.EXACT(“Information seeking behaviour”) *(Limit to peer-reviewed applied)*	4,299,298
	S3	ab(experience OR perception OR construct* OR perceiv*) OR MAINSUBJECT.EXACT(“Social construction”) *(Limit to peer-reviewed applied)*	1,842,495
	S4	ab(self* OR identity) OR (MJMESH.EXACT.EXPLODE(“Gender Identity: F.01.752.747.385.200”) OR MJMESH.EXACT.EXPLODE(“Gender Identity: F.01.393.446.250”) OR MJMESH.EXACT.EXPLODE(“Gender Identity: F.01.752.747.722.200”) OR MJMESH.EXACT.EXPLODE(“Gender Identity: F.02.739.794.793.200”)) OR MJMESH.EXACT.EXPLODE(“Self Concept”) OR (MAINSUBJECT.EXACT(“Self control”) OR MAINSUBJECT.EXACT(“Self image”) OR MAINSUBJECT.EXACT(“Self representation”) OR MAINSUBJECT.EXACT(“Self esteem”) OR MAINSUBJECT.EXACT(“Self expression”)) OR MAINSUBJECT.EXACT(“Self awareness”) OR MAINSUBJECT.EXACT(“Otherness”) OR MAINSUBJECT.EXACT(“Gender identity”) *(Limit to peer-reviewed applied)*	934,690
	S5	S1 AND S2 AND S3 AND S4 *(Limit to peer-reviewed applied)*	**13**
PubMed	#1	“vaginismus”[MeSH Terms]	13
	#2	“dyspareunia”[MeSH Terms]	235
	#3	(vaginismus(Title) OR dyspareunia(Title) OR vaginism(Title) OR “genito-pelvic pain/penetration disorder”[Title] OR GPPPD[Title] OR “penetration disorder”[Title] OR “vaginal pain”[Title])	157
	#4	(#1 OR #2 OR #3)	340
	#5	(“help seeking”(Abstract) OR help-seeking(Abstract) OR treatment(Abstract) OR “treatment seeking”(Abstract) OR treatment-seeking(Abstract) OR diagnosis(Abstract) OR information(Abstract) OR relief(Abstract) OR support(Abstract)	2,215,641
	#6	“help seeking behaviour”[MeSH Terms]	370
	#7	“information seeking behaviour”[MeSH Terms]	1081
	#8	(#5 OR #6 OR #7)	2,215,843
	#9	(experience(Abstract) OR perception(Abstract) OR construct*(Abstract) OR perceiv*(Abstract)	497,710
	#10	(self*[Abstract] OR identity(Abstract))	333,093
	#11	self concept[MeSH Terms]	16,898
	#12	“gender identity”[MeSH Terms]	2627
	#13	(#10 OR #11 OR #12)	339,862
	#14	(#4 AND #8 AND #9 AND #13)	**10**
Scopus		(TITLE ( vaginismus OR dyspareunia OR vaginism OR “genito-pelvic pain/penetration disorder” OR “penetration disorder” OR gpppd OR “vaginal pain”) AND TITLE-ABS-KEY (“Help seeking” OR help-seeking OR treatment OR treatment-seeking OR “treatment seeking” OR diagnosis OR information OR relief OR support) AND TITLE-ABS-KEY (experience OR perception OR construct* OR perceiv*) AND TITLE-ABS-KEY (self* OR identity)) AND ( LIMIT-TO ( LANGUAGE, “English”))	**36**

**Figure 1.**
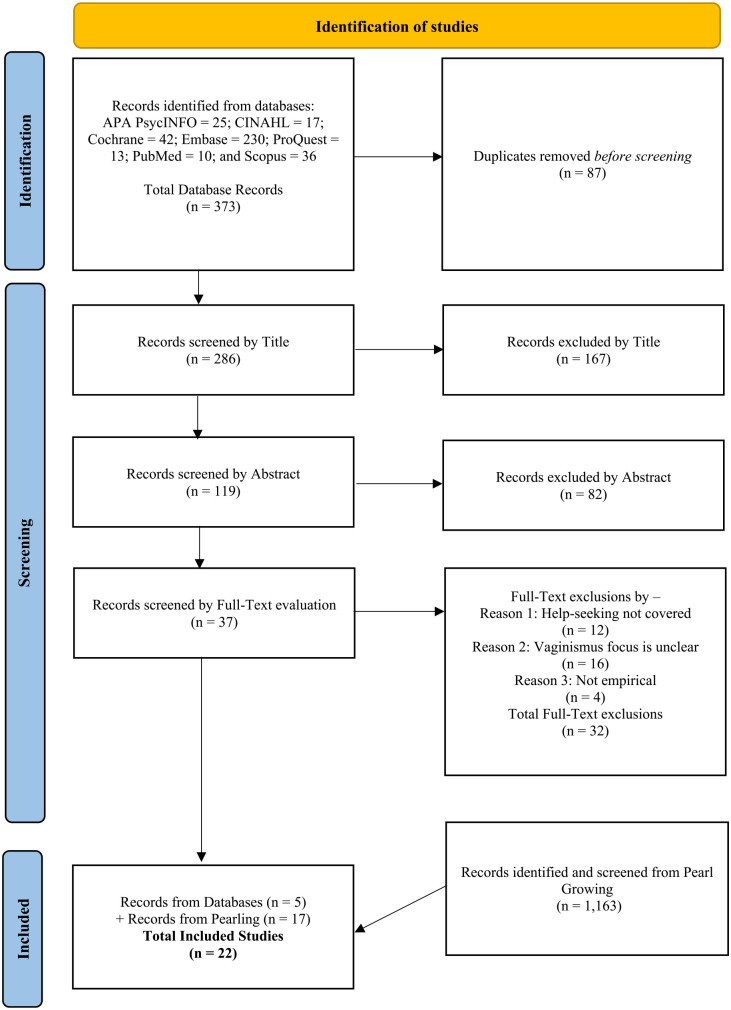
PRISMA flow chart outlining the article selection process.

In line with stage 2 of conducting an integrative review, it is recommended to use two or more search strategies to retrieve more relevant literature and reduce bias.^41^ Research has found that citation searching can uncover over half the studies relevant for a review^44^ and therefore be more comprehensive in identifying the relevant literature than database searches.^45^ Following this, an additional strategy of bi-directional citation searching (also known as pearl growing) was undertaken on the five studies by examining their reference list, and using Google Scholar to find and screen literature that it was ‘cited by’.^46^ This uncovered 17 more eligible studies. Altogether, 22 studies were identified as eligible and included in the review.^47–68^ See Figure 1 for a summary of the article selection process. Following PRISMA guidelines, all authors were involved in selecting the studies for inclusion. The authors discussed, and sometimes debated, which studies to include until consensus was reached.

### Quality assessment

As part of the data evaluation of stage three outlined by Cooper, the Mixed-Methods Appraisal Tool (MMAT) by Hong et al.^69^ was used to assess the methodological quality of the diverse study types of the 22 included articles. The MMAT uses five criteria to assess the quality of quantitative, qualitative, and mixed-method designs which enables the standardization of the quality assessment of diverse studies.^69^ While Hong et al’.s^69^ publication of the MMAT discouraged calculating a score for quality assessment, Hong et al.^70^ later recognized that it is problematic for reporting results. Hong et al.^70^ then suggested to score studies according to the number of MMAT quality criteria it meets out of 100%. Therefore, studies that meet: no criteria = 0%, 1 criterion = 20%, 2 criteria = 40%, 3 criteria = 60%, 4 criteria = 80%, and 5 criteria = 100%.^70^ A higher MMAT score indicates higher methodological quality.^70^ The first author initially assessed the included studies with the MMAT and then the second and third authors reviewed it and any disagreements were discussed until resolved with the results presented in Table 3. While 13 studies scored 80%–100%, three studies scored 60%. Due to scoring 40% or less, the remaining six studies were of lower methodological quality. Lower methodological quality can indicate a lack of rigour and an increased risk of bias for review findings. However, no studies were excluded based on their quality assessment by the MMAT to avoid missing any new findings in an under-researched area.

**Table 3.** MMAT quality assessment of the included studies.

Study	Study design	Methodological quality criteria	Responses			
			Yes	No	Unclear	Comments
Achour et al.^47^	Mixed methods	Is there an adequate rationale for using a mixed-methods design to address the research question?			X	
		Are the different components of the study effectively integrated to answer the research question?	X			
		Are the outputs of the integration of qualitative and quantitative components adequately interpreted?	X			
		Are divergences and inconsistencies between quantitative and qualitative results adequately addressed?		X		
		Do the different components of the study adhere to the quality criteria of each tradition of the methods involved?			X	It is unclear whether the nonresponse bias was low for the quantitative component
Akhavan-Taghavi et al.^48^	Quantitative descriptive	Is the sampling strategy relevant to address the research question?	X			
		Is the sample representative of the target population?	X			
		Are the measurements appropriate?	X			
		Is the risk of nonresponse bias low?	X			Since it was 115 consecutive clinical records, it is assumed that the nonresponse bias is low
		Is the statistical analysis appropriate to answer the research question?			X	This is not explained.
Bhatt et al.^49^	Qualitative	Is the qualitative approach appropriate to answer the research question?			X	There is no research question or aim clearly presented
		Are the qualitative data collection methods adequate to address the research question?			X	There is no research question or aim clearly presented
		Are the findings adequately derived from the data?	X			
		Is the interpretation of results sufficiently substantiated by data?	X			
		Is there coherence between qualitative data sources, collection, analysis, and interpretation?	X			
Drenth et al.^50^	Quantitative descriptive	Is the sampling strategy relevant to address the research question?	X			
		Is the sample representative of the target population?	X			
		Are the measurements appropriate?	X			
		Is the risk of nonresponse bias low?	X			
		Is the statistical analysis appropriate to answer the research question?	X			
Engman et al.^51^	Quantitative descriptive	Is the sampling strategy relevant to address the research question?	X			
		Is the sample representative of the target population?	X			
		Are the measurements appropriate?	X			
		Is the risk of nonresponse bias low?	X			
		Is the statistical analysis appropriate to answer the research question?	X			
Fageeh^52^	Qualitative	Is the qualitative approach appropriate to answer the research question?	X			
		Are the qualitative data collection methods adequate to address the research question?	X			
		Are the findings adequately derived from the data?	X			
		Is the interpretation of results sufficiently substantiated by data?		X		The Discussion section is not linked to the presented data
		Is there coherence between qualitative data sources, collection, analysis and interpretation?		X		There is lack of coherence because the interpretation in the Discussion is not linked to the data sources
Fejza et al.^53^	Qualitative	Is the qualitative approach appropriate to answer the research question?	X			
		Are the qualitative data collection methods adequate to address the research question?		X		There is no clear methods section to explain or justify data collection
		Are the findings adequately derived from the data?			X	There is lack of coherence because the interpretation in the Discussion is not linked to the data sources
		Is the interpretation of results sufficiently substantiated by data?		X		There is no Discussion or any other clear section to interpret the Results
		Is there coherence between qualitative data sources, collection, analysis and interpretation?		X		There is no clearly marked Results or Discussion section to cohere the data, collection, analysis and interpretation
Kurban et al.^54^	Quantitative descriptive	Is the sampling strategy relevant to address the research question?	X			
		Is the sample representative of the target population?	X			
		Are the measurements appropriate?	X			
		Is the risk of nonresponse bias low?			X	
		Is the statistical analysis appropriate to answer the research question?	X			
Macey et al.^55^	Qualitative	Is the qualitative approach appropriate to answer the research question?	X			
		Are the qualitative data collection methods adequate to address the research question?	X			
		Are the findings adequately derived from the data?	X			
		Is the interpretation of results sufficiently substantiated by data?	X			
		Is there coherence between qualitative data sources, collection, analysis and interpretation?	X			
Muammar et al.^56^	Quantitative descriptive	Is the sampling strategy relevant to address the research question?	X			
		Is the sample representative of the target population?	X			
		Are the measurements appropriate?	X			
		Is the risk of nonresponse bias low?		X		The information on nonrespondents has not been provided to assess the risk of bias
		Is the statistical analysis appropriate to answer the research question?	X			Sample size not justified
Ogden and Ward^57^	Mixed methods	Is there an adequate rationale for using a mixed-methods design to address the research question?	X			
		Are the different components of the study effectively integrated to answer the research question?	X			
		Are the outputs of the integration of qualitative and quantitative components adequately interpreted?	X			
		Are divergences and inconsistencies between quantitative and qualitative results adequately addressed?		X		
		Do the different components of the study adhere to the quality criteria of each tradition of the methods involved?	X			
O’Sullivan^58^	Qualitative	Is the qualitative approach appropriate to answer the research question?			X	There is no research question or aim clearly presented. This may be due to the age of the research
		Are the qualitative data collection methods adequate to address the research question?			X	There is no research question or aim clearly presented. This may be due to the age of the research
		Are the findings adequately derived from the data?	X			
		Is the interpretation of results sufficiently substantiated by data?	X			
		Is there coherence between qualitative data sources, collection, analysis and interpretation?	X			
Ramli et al.^59^	Qualitative	Is the qualitative approach appropriate to answer the research question?	X			
		Are the qualitative data collection methods adequate to address the research question?			X	No justification provided for the chosen number of participants
		Are the findings adequately derived from the data?		X		Only the first paragraph of the discussion was derived from the data
		Is the interpretation of results sufficiently substantiated by data?			X	Data presented as cases is not sufficiently analysed nor interpreted in the Discussion
		Is there coherence between qualitative data sources, collection, analysis and interpretation?			X	The way that the data were collected and analysed is not clear and therefore does not clearly cohere with the interpretation of findings
Reissing^60^	Quantitative descriptive	Is the sampling strategy relevant to address the research question?	X			
		Is the sample representative of the target population?	X			
		Are the measurements appropriate?	X			
		Is the risk of nonresponse bias low?	X			
		Is the statistical analysis appropriate to answer the research question?			X	The statistical analysis used is neither clearly stated nor justified
Scholl^61^	Quantitative non-randomized	Are the participants representative of the target population?	X			
		Are measurements appropriate regarding both the outcome and intervention (or exposure)?	X			
		Are there complete outcome data?	X			
		Are the confounders accounted for in the design and analysis?		X		
		During the study period, is the intervention administered (or exposure occurred) as intended?	X			
Stelko^62^	Qualitative	Is the qualitative approach appropriate to answer the research question?	X			
		Are the qualitative data collection methods adequate to address the research question?	X			
		Are the findings adequately derived from the data?	X			
		Is the interpretation of results sufficiently substantiated by data?	X			
		Is there coherence between qualitative data sources, collection, analysis, and interpretation?	X			
Svedhem et al.^63^	Qualitative	Appropriate approach to answer research question	X			
		Adequate data collection methods	X			
		Findings are adequately derived from data	X			
		Interpretations of results substantiated by data	X			
		Coherence between qualitative data sources, collection, analysis, and interpretation	X			
Thorpe et al.^64^	Qualitative	Appropriate approach to answer research question	X			
		Adequate data collection methods	X			
		Findings are adequately derived from data	X			
		Interpretations of results substantiated by data	X			
		Coherence between qualitative data sources, collection, analysis, and interpretation	X			
Tulla et al.^65^	Qualitative	Appropriate approach to answer research question	X			
		Adequate data collection methods	X			
		Findings are adequately derived from data			X	
		Interpretations of results substantiated by data		X		The discussion is largely speculative rather than based on the findings
		Coherence between qualitative data sources, collection, analysis, and interpretation		X		Several points in the discussion are not based on the findings from the case study
Ward and Ogden^66^	Mixed methods	Is there an adequate rationale for using a mixed-methods design to address the research question?			X	The study states that quantitative and qualitative methods are used. But there is no explicit statement that mixed methods are used to justify its rationale
		Are the different components of the study effectively integrated to answer the research question?	X			
		Are the outputs of the integration of qualitative and quantitative components adequately interpreted?	X			
		Are divergences and inconsistencies between quantitative and qualitative results adequately addressed?	X			
		Do the different components of the study adhere to the quality criteria of each tradition of the methods involved?	X			
Zgueb et al.^67^	Mixed methods	Is there an adequate rationale for using a mixed-methods design to address the research question?		X		No mention of using mixed methods. No explicit rationale provided for the use of mixed methods
		Are the different components of the study effectively integrated to answer the research question?		X		The quantitative assessment was not adequately covered or integrated
		Are the outputs of the integration of qualitative and quantitative components adequately interpreted?		X		The quantitative assessment was not adequately integrated to be interpreted
		Are divergences and inconsistencies between quantitative and qualitative results adequately addressed?		X		Any divergences and inconsistencies are not mentioned or explained
		Do the different components of the study adhere to the quality criteria of each tradition of the methods involved?		X		The statistical analysis used is not justified
Zulfikaroglu^68^	Mixed methods	Is there an adequate rationale for using a mixed-methods design to address the research question?		X		No mention of using mixed methods. No explicit rationale is provided for the use of mixed methods
		Are the different components of the study effectively integrated to answer the research question?			X	
		Are the outputs of the integration of qualitative and quantitative components adequately interpreted?	X			
		Are divergences and inconsistencies between quantitative and qualitative results adequately addressed?		X		
		Do the different components of the study adhere to the quality criteria of each tradition of the methods involved?		X		

### Data analysis

While commonly used to summarize qualitative research, thematic analysis can also be used to synthesize diverse quantitative, qualitative, and mixed-method studies for analysis,^71,72^ including in integrated reviews.^73^ Since quantitative and qualitative data can address the same research phenomena, these different types of data can be transformed into the same format to be organized and analysed.^74^ Therefore, to follow stage four of data analysis in Cooper’s framework, Dwyer^73^ recommends to use Braun and Clarke’s^75^ six-phase approach of thematic analysis in integrative reviews. This review uses Braun and Clarke’s^73,75^ thematic approach to extract, organize, and analyse the findings from the included studies.

First, familiarization with the qualitative and quantitative data occurred by reading the Results, Discussion, and Conclusion sections of the included studies several times and noting initial ideas for coding.^75^ Quantitative studies were ‘qualitized’, as Sandelowski et al.^74^ call it, by repeatedly reading and taking notes on the descriptive and inferential statistics and summarized quantitative results articulated in the findings. Second, initial codes from the data were generated.^75^ Third, themes were searched using Quirkos, a computer-assisted qualitative data analysis software (CAQDAS), as an organizational tool to extract data to answer the research question and visually sort codes into themes.^75^ For the fourth and fifth phase, the themes were reviewed and refined with appropriate subthemes.^75^ A narrative report of the themes is presented below for the sixth phase.^75^ According to Toronto,^38^ statistical synthesis methods such as meta-analysis are not included in integrative reviews.

## Results

### Sample

Out of the 22 included studies, seven studies had quantitative study designs, 10 were qualitative studies, and five used mixed-method designs. The 22 studies included 1671 women’s help-seeking experiences for vaginismus. The sample varied to include women who either currently or formerly had vaginismus, and those who had primary and secondary vaginismus. These women interacted with a range of health professionals and tried various treatments. The participants ranged from 18 to 71 years of age in 21 of the 22 studies. The remaining study by Stelko^62^ did not disclose participants’ ages. The sample represented women from Saudi Arabia, Iran, Tunisia, Turkey, India, Netherlands, Sweden, Australia, the United States, the United Kingdom, and Malaysia. Only four included studies by Ward and Ogden,^66^ Ogden and Ward,^57^ Svedhem et al.,^63^ and Thorpe et al.^64^ mentioned participants’ sexual orientation. Stelko^62^ discussed heterosexuality at length but the sexual orientation of participants was not stated. No studies indicated gender diversity. The characteristics of the included studies, including methods used, are summarized in Table 4.

**Table 4.** Characteristics of the included studies in the integrative review.

No.	Authors/year	Condition	Country/city	Theoretical approach	Sample size	Study design	Methods	Analytical approach
1.	Achour et al.^47^	Vaginismus and pregnancy	Tunisia	Not stated	20	Mixed methods^€^	Interviews with standardized questionnaire and STAI	Descriptive analysis
2.	Akhavan-Taghavi et al.^48^	Vaginismus	Tehran, Iran	Not stated	115	Quantitative	Data from clinical records extracted using a standard questionnaire	Descriptive analysis
3.	Bhatt et al.^49^	Vaginismus	Gujarat, India	Not stated	25	Qualitative	Retrospective study	Not stated
4.	Drenth et al.^50^	Vaginismus	Groningen, the Netherlands	Not stated	49	Quantitative	Retrospective study	Descriptive statistics
5.	Engman et al.^51^	Primary or Secondary, and Partial or Total Vaginismus	Linköping, Sweden	Not stated	44	Quantitative	Questionnaire and therapy records	Pre- and post-intervention comparisons (Pearson Chi-squared test and Wilcoxon’s rank sum test)
6.	Fageeh^52^	Vaginismus	Jeddah, Saudi Arabia	Not stated	15	Qualitative	Interviews	Descriptive analysis
7.	Fejza et al.^53^	Vaginismus	Not stated (assumed to be Kosovo^Ω^)	Not stated	3	Qualitative	Case series	Not stated
8.	Kurban et al.^54^	Primary vaginismus	Ankara, Turkey	Not stated	482	Quantitative	Questionnaire	Descriptive statistics
9.	Macey et al.^55^	Lifelong or acquired vaginismus	Online and Nottingham, East Midlands, the United Kingdom^µ^	Not stated	13	Qualitative	Interviews	Thematic analysis
10.	Muammar et al.^56^	Vaginismus	Saudi Arabia	Not stated	100	Quantitative	Retrospective descriptive study	Pre- and post-intervention comparisons (Chi-squared tests)
11.	Ogden and Ward^57^	Former or current vaginismus	The United Kingdom, Australia, and United States	Not stated	89	Mixed methods	Surveys with free responses	Quantitative descriptive statistics and thematic analysis^¥^
12.	O’Sullivan^58^	Vaginismus	Ireland	Not stated	23	Qualitative	Observational study	Qualitative categories and descriptive statistics^¥^
13.	Ramli et al.^59^	Vaginismus	Malaysia	Not stated	3	Qualitative	Case series	Not stated
14.	Reissing^60^	Lifelong or acquired vaginismus	Online	N/A	168	Quantitative	Surveys	Between lifelong- and acquired-vaginismus group comparisons (Chi-squared tests)
15.	Scholl^61^	Vaginismus	New York, United States^µ^	Not stated	24	Quantitative	Non-randomized study^£^	Non-parametric two-tailed Mann-Whitney test
16.	Stelko^62^	Primary vaginismus	Online – United States, European countries, and Israel	Not stated	12	Qualitative	In-depth interviews	Not stated
17.	Svedhem et al.^63^	Vaginismus	Sweden	Grounded theory	8	Qualitative	Semi-structured interviews	Interpretative phenomenological analysis (IPA)
18.	Thorpe et al.^64^	Vaginismus	Southern United States	Not stated	1^β^	Qualitative	Semi-structured interviews	Constructivist grounded theory
19.	Tulla et al.^65^	Vaginismus	New York, United States^µ^	Not stated	1	Qualitative	Case study	Not stated
20.	Ward and Ogden^66^	Former or current vaginismus	The United Kingdom, Australia, and United States	N/A	89^±^	Mixed methods	Surveys with free responses	Descriptive statistics, between former- and current-vaginismus groups comparisons (two-tailed t-tests), and discourse analysis
21.	Zgueb et al.^67^	Vaginismus	Not stated (assumed to be Tunisia^Ω^)	N/A	4	Mixed methods	Case series	N/A
22.	Zulfikaroglu^68^	Vaginismus	Ankara, Turkey	N/A	472	Mixed methods	Cross-sectional methodology, interview questionnaire, and gynaecological examination	Descriptive analysis

IPA: interpretative phenomenological analysis.

€ Paper did not explicitly state mixed methods – this was assumed from the manuscript.

Ω Assumed city/country as this information was not provided.

µ City/country was inferred from given information.

¥ Paper did not explicitly state analytical methods – this was assumed from the manuscript.

£ Paper did not explicitly state it was a non-randomized study – this was assumed from the manuscript.

± Ogden and Ward^57^ and Ward and Ogden^66^ used the same 89 participants in their studies which were only counted once in the sample total.

β This study included 25 participants with various sexual pain issues. Only the data from one participant with vaginismus were extracted for this review.

### Thematic analysis results

Four major themes emerged: *Help-seeking process, Medical management, Help-seeking and sense of self, and Holistic care recommendations from the included studies*. The major themes and relevant subthemes are detailed below as part of stage five outlined by Cooper^39^ for the presentation of the integrative review findings. References are presented next to the key findings.

#### Theme 1: Help-seeking process

All the included studies described women’s experiences of seeking help for vaginismus in some way. Their experiences reflected both internal and external factors that influenced their treatment journeys. This included women’s *Motivations* to seek help and varied experiences with *Health Professionals*.

##### Motivations

Women with vaginismus were largely motivated by heterosexual and heteronormative expectations of womanhood to seek help. Women’s motivations included wanting to conceive a child, fears of not being able to have a long-term relationship or losing their partner, and wanting to achieve penis-in-vagina (PIV) sex.^47,49–51,53,54,56,58–60,63,65,67^ Women who were specifically motivated to treat their vaginismus to conceive a child were found to achieve treatment success faster than those who were only motivated to achieve PIV sex.^50,61^ However, it was also found that some women with vaginismus, who had not yet achieved PIV sex with treatment, used artificial or self-insemination strategies for conception.^47,50,59,60,63,65,67^ Self-insemination strategies included ‘splash pregnancy’ wherein there is no PIV penetration and instead ejaculation at the vaginal opening propels the sperm to the ovaries.^59^

Only some women with vaginismus were motivated to seek help for other reasons, such as being able to insert tampons and to have examinations without anxiety or fear.^51,60,63^ For many women, previous, or anticipated, bad experiences with the healthcare system demotivated them from seeking help for their vaginismus.^47,55,57,61^ Often, women had to negotiate their personal reservations and the social taboo of discussing sexual matters before they could feel motivated to begin to seek help.^47,53,55,58,59,62,64^ Consequently, it frequently took women 1–3+ years to begin seeking help,^48–50,53,54^ and up to 5–10 years in some cases.^52,59^

##### Health professionals

Women consulted a range of health professionals for their vaginismus. They found it helpful when health professionals were knowledgeable, emotionally supportive, and listened non-judgementally.^49,57,64^ Health professionals were unhelpful when they lacked understanding, did not appreciate or appropriately respond to women’s concerns pertaining to their vaginismus, or gave generic verbal advice such as telling women to ‘relax’.^47,48,55,56,62,64^ If women perceived having negative experiences with health professionals who did not take them seriously or made inappropriate comments, they stopped help-seeking for vaginismus and sometimes took years to seek help again.^47,50,55^

Women valued health professionals who had the specialist expertise to offer specific advice on treatments. In the countries of the United Kingdom, Australia, and the United States, women with vaginismus were most likely to first consult general practitioners to seek help and treatment.^55,57,60^ In the studies based in Tunisia, Iran, Saudi Arabia, and Turkey, specialist health professionals were more often consulted by women for vaginismus.^47,48,56,67,68^

#### Theme 2: Medical management

All the reviewed studies explored women’s different experiences to gain *Diagnosis* and/or undertake treatment *Interventions*. Some women indicated that there were difficulties in gaining a diagnosis of vaginismus. However, others also described how interventions were helpful.

##### Diagnosis

The studies indicated that women often consulted several sources, which could take years, to finally receive a diagnosis of vaginismus.^47,50,53,55,57,60^ Many women either had difficulty or were unable to complete physical examination which informed their diagnosis.^47,55,58,59,65,67^ Some women had received a prior diagnosis of provoked vestibulodynia, generalized vulvodynia, or a narrowed or thickened hymen.^48,54,56,60,68^ Many women sought help specifically for infertility and were diagnosed with vaginismus.^47,49,58,59,61^ The partners of some women with vaginismus were also diagnosed with male sexual dysfunctions.^48,49,52,58,59,66,68^

##### Interventions

Women attempted various interventions, often over several years. Common conventional and conservative treatments, which did not include invasive procedures, that women tried were vaginal trainers, pelvic floor exercises such as Kegels, relaxation, sensate focus exercises, sex education, various psychotherapies, cognitive behavioural therapy (CBT), and exposure therapy.^49,51–56,58–60,62^ Interventions had to address myths that women believed such as having a narrow vagina, pain and bleeding after having sex for the first time, and vaginal rupture with sex.^49,54,59,66^ Women’s partners often, but not always, participated in treatment such as therapy, physical touch exercises, or lending their fingers for vaginal insertion and desensitization with varying results.^53,55,57,65,67^

Women had varied responses to using vaginal trainers. While many women found vaginal trainers helpful,^49,55,62,65^ others needed more instructions to appropriately use trainers,^55^ or required complementary treatments such as lubricant,^62^ topical anaesthetic,^52^ or escitalopram.^56^ Women found it harder to progress through conservative treatment when they had more severe vaginismus.^52,54^ A few of these women used medical aids to facilitate conservative treatment (such as Botox injections).^52,56^ Some women were reported to have received hymenotomy which often did not treat symptoms.^48,53,54,68^ A few women insisted on, and received, a hymenotomy despite health professionals advising them that it was not necessary.^53^

The authors of the studies based in Turkey, Saudi Arabia, Kosovo, and Tunisia described the societal culture as religious and viewing vaginismus as taboo.^53,56,67,68^ In these studies, women were reported as consulting faith healers or traditional non-academic medicine for their vaginismus.^53,56,67,68^ Moreover, treatment was adapted to suit the cultural context in more conservative countries.^53,62,67^ For example, religious figure heads were involved in CBT interventions to reframe the patient’s thinking,^67^ or women made their own vaginal trainers in countries where they were not allowed to buy clinical vaginal trainers due to religious reasons.^62^ Also, in more culturally conservative countries, such as the studies based in Tunisia, Iran, Kosovo, and Turkey, women referred to not receiving education from their family, hiding their vaginismus from relatives, or feeling pressure from family to conceive a child when they got married.^47,48,53,54,67^

#### Theme 3: Help-seeking and sense of self

There was a lack of explicit discussion of how women’s help-seeking for vaginismus shaped their constructions of self. Hence, this theme describes the limited findings from three included studies which report on women’s experiences of self in the context of living with their vaginismus.^75^ Engman et al.^51^ briefly discussed a correlation between women with vaginismus who completed CBT and a reported higher sense of self-worth as women and human beings. However, Engman et al.^51^ also noted that they were uncertain whether the reported increase in self-worth was a result of CBT treatment because no such study exists to confirm this correlation. Ogden and Ward^57^ explored how experiencing vaginismus influences women’s self-perception. Stelko^62^ considers women’s experiences of vaginismus in relation to heteronormativity. While Engman et al.^51^ and Ogden and Ward^57^ more directly reported on the self, Stelko^62^ implicitly discussed the self in relation to women’s perceptions of heteronormativity. However, these three studies did not explicitly consider how the process of help-seeking, specifically interactions with the healthcare system and other avenues to gain information, diagnosis, and treatment, impacts women’s constructions of self.

#### Theme 4: Holistic care recommendations from the included studies

The recommendations from nineteen included studies were centred around improving women’s healthcare experiences. Women and health professionals having congruent expectations towards the medical management of vaginismus may determine productive help-seeking experiences.^47,49,52,55,57,61^ Incongruent beliefs between patients and health professionals may foreshadow negative and varied help-seeking experiences for women with vaginismus.^47,57^ Therefore, health professionals should strive to have “a mutual understanding” with their patients regarding experiences of vaginismus and “expectations about therapeutic interventions” (p. 29).^57^

Women may not initiate a discussion about vaginismus due to the stigma associated with sexual health.^47,53,63^ Those with vaginismus indicated that they would prefer health professionals to initiate discussions regarding their sexual health rather than bring up sexual concerns themselves due to it making them feel uncomfortable.^47,50,53,63^ Health professionals who are often the initial contact for vaginismus should be trained in sexual health to better treat women with the condition.^54,56,65^ There should be a multidisciplinary health professional team to support the varied treatment needs of women with vaginismus.^47,56,59,60,65^ Also, the approach to treat vaginismus should be adapted to suit the cultural context of the patients.^47,53,55,56,67,68^ In predominantly Muslim countries, given the closeness and/or importance of the family, treatment should educate family members to better support women with vaginismus.^48,67,68^

## Discussion

This review found that while women with vaginismus have varied experiences of seeking help, there are also commonalities. Common unhelpful actions of health professionals were to not appropriately respond to women’s raised concerns or provide generic verbal advice which fostered negative help-seeking experiences for women. Such unhelpful actions cannot be assumed as a sign of uncaring health professionals. Rather, the review’s findings suggest that health professionals, including doctors, are lacking knowledge to support those with vaginismus and recognize symptoms of the condition. This aligns with Pacik’s^3^ contention that vaginismus is not sufficiently taught in medical schools or discussed in medical meetings. In fact, in a study by Auwad and Hagi,^76^ 73% of Arab gynaecologists, 47% of British Society of Urogynaecology (BSUG) gynaecologists, and 23% of the American Urogynaecologic Society (AUGS) gynaecologists reported themselves as being unsatisfied with their training in female sexual dysfunction. Therefore, it is important that all health professionals, especially health professionals who tend to be the first port of healthcare, are trained in future to be better versed in female sexual dysfunctions. This may help to minimize the number of referrals that those with vaginismus need to gain a diagnosis.

Women with vaginismus appeared to be predominantly motivated to seek help to meet the heterosexual and heteronormative expectations for women. Put differently, women with vaginismus seemed driven to seek help by a desire to achieve PIV sexual intercourse, which is also known as the ‘coital imperative’.^77,78^ In the studies based in Tunisia, India, Kosovo, Saudi Arabia, Malaysia, and Turkey, women’s coital imperative was often primarily driven by societal expectations to become a mother.^47,49,53,56,59,68^ While women in the studies focused in the Netherlands, Sweden, the United States, and European countries were motivated to seek help for vaginismus to become mothers, many were also driven by a need to sexually satisfy their partners.^50,51,60,62,63^ Such desires to become a mother and sexually please male partners through PIV sex are part of the embodied expectations held towards heterosexual women.^79^

The studies did not directly discuss how women’s help-seeking for vaginismus impacted their sense of self. Nonetheless, information gathered within this review suggests that women’s help-seeking for vaginismus shaped their perceived sense of self with respect to conforming or deviating from heteronormative femininity. The ideals of heteronormative femininity dictate that ‘a good woman’ is: receptive to the penis for the coital imperative; a man’s partner; and a mother.^80^ Since women with vaginismus may not be able to achieve the aforementioned, they often feel like their ‘self-image of femininity’ has been jeopardized and have trouble ‘believing in themselves as women’ (p. 12).^33^ This can drive women to not believe in themselves, which can exacerbate their struggles to find help or follow treatment.

Strategies should be developed and employed by health professionals to ‘redo’ heteronormative femininity by normalizing non-penetrative sex.^81,82^ This can strengthen women’s sense of self to believe in themselves as capable women to persevere even when their treatment-seeking journey is trying. However, the heterosexual and heteronormative motivations of those with vaginismus in the included studies cannot be generalized to all people with the condition. These findings may be skewed by the generally heterosexual orientation of participants in the studies. For instance, those with diverse sexual and gender identities often do not subscribe to heteronormative femininity, or relate to the embodied expectations held towards heterosexual women.

Given that the locations of the included studies were based in some religious and other non-religious countries, key cultural differences between women’s help-seeking experiences for vaginismus were highlighted. The prevalence of vaginismus in religious countries has been reported to be higher.^6,17,67,83,84^ In religious societies where women are very close to family, trusted religious figures and family members may need to be involved in treatment to foster the appropriate support and positive interactions to help strengthen women’s sense of self to overcome vaginismus.^48,67,68^ Moreover, Stelko^62^ referred to how women in her study had to make vaginal trainers due to not being able to buy it in certain countries due to religious reasons. Conventional vaginismus treatments focused on vaginal insertion and physical touch may be taboo in the religious cultures of certain countries.^67^ Therefore, conventional treatment approaches for vaginismus and professional practice should be adapted to suit women’s cultural context to foster positive help-seeking experiences for them.^21,67^

Six out of the seven studies based in the multicultural Western countries of Australia, the United States, and the United Kingdom did not articulate that attempts were made to recruit participants from diverse ethnic backgrounds.^55,57,60,61,65,66^ While Thorpe et al.^64^ focused on Black women, the data from one participant in their study which was extracted into this integrated review were not enough to inform results on ethnically diverse women’s experiences of vaginismus and its impact on their constructions of self. It cannot be assumed that all the participants from the other six studies identified as White. However, future research on this topic should strive to include participants from diverse non-White backgrounds, especially in multicultural Western countries. This can help to generate data to improve the unique help-seeking experiences for women with vaginismus from culturally diverse backgrounds when navigating Western healthcare systems.

The fact that none of the included studies explicitly discussed how help-seeking for vaginismus impacts women’s sense of self highlights an epistemological gap in the literature. It has been overlooked that help-seeking impacts women’s strength of the therapeutic alliance and constructions of sense of self in ways that can have implications on their treatment journeys for vaginismus. This should be investigated in future research to improve health outcomes for those who receive a vaginismus diagnosis.

### Limitations

In line with stage five of Cooper’s framework, the limitations of the integrated review are examined. The participants in the included studies were described exclusively as women. The sexual orientation of participants was missing from 18 studies. Therefore, the unique help-seeking experiences of those who do not identify as women or heterosexual could not be represented in this review. Only three studies^55,57,60^ focused on help-seeking in its research aim or objective which may reflect that the concept and term ‘help-seeking’ is not universally used. Six included studies that were of low methodological quality according to the MMAT could potentially have a high risk of bias, which reduces the trustworthiness of the review findings. Future studies on women’s experiences of vaginismus need to strive to better meet its methodological criteria to increase the number of quality studies in an under-researched area. Some of this review’s findings are informed by included studies that have very small sample sizes which poses a risk of participant selection bias. Fourteen included studies lacked a clearly stated theoretical approach. This presents issues for the interpretation of findings, and the potential of transferability because there is no guide to conceptualize how a study applies to broader contexts.^85^ Unpublished and non-peer-reviewed literature, such as master's and doctoral theses, were excluded from the search. However, the retrieval and analysis of unpublished dissertations is time-consuming and laborious, and the benefit of including dissertations in reviews is minimal as they are unlikely to change the conclusions.^86^

## Conclusion

This is the first integrative review to describe mixed methods of evidence to examine women’s help-seeking experiences for their vaginismus and its impact on their constructions of self. The review found that women continue to face difficulties in seeking and receiving help for vaginismus. Conventional treatment approaches for vaginismus should be amended to suit women’s cultural context to improve their help-seeking outcomes. The review also identified an epistemological gap in the literature regarding how women’s help-seeking experiences for their vaginismus impact their constructions of self, which highlights it as an under-researched area. This review of evidence uncovers an important inferred correlation between women’s help-seeking for vaginismus shaping their construction of a sense of self in relation to heteronormative femininity. Future research should not be limited to heterosexual women but include the experiences of those with vaginismus who have diverse gender identities. The experiences of those from culturally and ethnically diverse backgrounds, especially in multicultural Western countries, should be examined. Research should focus on people’s help-seeking for vaginismus and its impact on their sense of self to provide insight into how it can determine their responses to treatment. Such findings can be used in the future for people with vaginismus, health professionals, and in policy to understand how to better support those with vaginismus to seek help and construct a positive sense of self to persevere with treatment regimens.

## Supplemental Material

sj-docx-1-whe-10.1177_17455057231199383 – Supplemental material for The experiences of women seeking help for vaginismus and its impact on their sense of self: An integrative review Click here for additional data file. Supplemental material, sj-docx-1-whe-10.1177_17455057231199383 for The experiences of women seeking help for vaginismus and its impact on their sense of self: An integrative review by Rashmi Pithavadian, Jane Chalmers and Tinashe Dune in Women’s Health

## Declarations

**Ethics approval and consent to participate:** Ethics approval and consent was not required for this research because it did not involve human participants. Published information that is available online was used as the dataset.

**Consent for publication:** Not applicable

**Availability of data and materials:** Database searches have been provided as a table for replication of search results. Any further information pertaining to data will be available upon reasonable request from the corresponding author’s email address (R.Pithavadian@westernsydney.edu.au).
